# An UPLC-MS/MS Method for Determination of Osimertinib in Rat Plasma: Application to Investigating the Effect of Ginsenoside Rg3 on the Pharmacokinetics of Osimertinib

**DOI:** 10.1155/2020/8814214

**Published:** 2020-12-28

**Authors:** Zhenzhen Ying, Jingyao Wei, Ruijuan Liu, Fang Zhao, Yifang Yu, Xin Tian

**Affiliations:** ^1^Department of Pharmacy, The First Affiliated Hospital of Zhengzhou University, Zhengzhou 450052, China; ^2^Henan Key Laboratory of Precision Clinical Pharmacy, Zhengzhou 450052, China

## Abstract

Osimertinib is a novel oral, potent, and irreversible epidermal growth factor receptor tyrosine kinase inhibitor (EGFR-TKI) for treatment of advanced T790M mutation-positive advanced non-small cell lung cancer, which is commonly combined with ginsenoside Rg3 in clinic to enhance the efficacy and minimize adverse reactions. In the present study, a highly sensitive UPLC-MS/MS method was established and validated for analysis of osimertinib in rat plasma according to US FDA guideline. Separation was performed on a C18 (2.1 × 50 mm, 2.6 *μ*m) column using a gradient elution of ammonium formate (10 mM) with 0.1% formic acid buffer (A) and ACN (B) at a flow rate of 0.2 mL/min. Detection was carried out on a triple quadrupole tandem mass spectrometer equipped with electrospray ionization in the MRM mode. The method was validated over a concentration range of 1–400 ng/mL for osimertinib. The intra- and interday accuracy and precision values were within ±15%. No significant degradation occurred under the experimental conditions in stability assays. There was a further investigation on the effects of multiple doses of ginsenoside Rg3 on the pharmacokinetics of osimertinib in rats for the first time. The results implied that osimertinib exhibited a slow absorption and moderate-rate elimination in rats following oral administration. Coadministeration with ginsenoside Rg3 (5 mg/kg, 7 days, *i.g.*) may have no effect on the pharmacokinetics of osimertinib in rats. The results provide a reference for the clinical concomitant medications of Rg3 and osimertinib.

## 1. Introduction

Osimertinib (AZD9291), the first third-generation oral potent and irreversible epidermal growth factor receptor tyrosine kinase inhibitor (EGFR-TKI), was approved by US-FDA and NMPA for first-line treatment of advanced non-small cell lung cancer (NSCLC) patients with acquired metastatic EGFR T790M mutation [1, 2]. Pharmacokinetic results shows that the absorption of osimertinib in rats was slow with the *T*_max_ values about 4.48 h, and the elimination might be slow (*t*_1/2_ and MRT values were estimated to be 14.96 and 14.51 h) [3]. Preclinical studies demonstrate that osimertinib is principally metabolized by cytochrome P450 (CYP3A), and AZ5104 and AZ7550 are the two circulating active metabolites. In vitro studies also describe osimertinib as the substrate of P-gp and BCRP [3]. Hence, the pharmacokinetic profiles and therapy effect of osimertinib could be significantly altered when coadministered with other medications. A phase I study found that the exposure (AUC) of osimertinib increased by 24% when coadministered with itraconazole (strong CYP3A4 inhibitor) and decreased by 78% when coadministered with rifampicin (CYP3A4 inducer) in patients with advanced NSCLC [4].

Ginsenoside Rg3, the compound extracted from the Chinese herb Panax ginseng, proved to inhibit the growth, invasion, and metastasis of several kinds of malignancies, including lung cancer, breast cancer, ovarian cancer, glioma, and leukemia, and enhance the efficacy of chemotherapy. Kim et al. reported that Shenyi capsule (the main active ingredient is ginsenoside Rg3) could improve the life span of NSCLC patients by improving the immune function and antitumor angiogenesis [5]. Meta-analyses demonstrated that Shenyi capsule plus chemotherapy could increase incidence of short-term efficacy and improve the quality of life and survival rate of NSCLC patients compared with chemotherapy alone [6]. However, many literatures reported that ginsenoside could modulate CYP450s and transporters [7–10]. Malati et al. found that the exposure of midazolam when orally administered via the gastrointestinal tract for 28 days in twelve healthy volunteers was significantly reduced, and the reason may be that the gastrointestinal tract induced CYP3A activity [9]. Lei-Qiong Yang reported that ginsenoside Rg3 significantly enhanced the oral bioavailability of paclitaxel in rats and improved antitumor activity in nude mice via inhibiting P-glycoprotein (P-gp) [11]. Dou et al. demonstrated that Rg3 could induce MRP1 to attenuate NAPQI-induced toxicity by activating Nrf2 [12]. In case of adverse reactions and inadequate dosages, drug interactions should be evaluated, when Rg3 and TKIs are comedicated.

An appropriate analytical method should be established for evaluating the drug interaction between Rg3 and osimertinib. However, to the best of our knowledge, there were only two published articles about liquid chromatography-tandem mass spectrometry (LC-MS/MS) methods for determination of osimertinib in rat plasma [13, 14]. There were some drawbacks in these methods such as being time-consuming, tedious sample preparation, and high sample volume (100 *μ*L), which was not adequate for pharmacokinetic studies. Therefore, this paper describes the development and validation of UPLC-MS/MS method for the quantitative analysis of osimertinib in rat plasma, using nilotinib as an internal standard. Samples were pretreated by an optimized protein precipitation method with acetonitrile (ACN) as the precipitant. This method provides a simple, sensitive, fast, and accurate method for the quantitative analysis of osimertinib. Meanwhile, this method was successfully applied for the drug-interaction pharmacokinetic study of osimertinib in rats after oral administration.

## 2. Experimental

### 2.1. Chemical and Reagents

**Figure 1:**
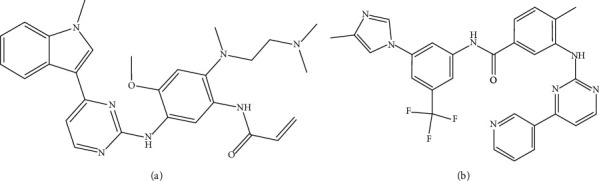
The chemical structures of osimertinib (a) and nilotinib (IS) (b).

Osimertinib (purity ≥ 99.95%) was purchased from MedChemExpress Co., Ltd. (Shanghai, China). Nilotinib (purity ≥ 99%) was procured from Aladdin Co., Ltd. (Shanghai). Shenyi capsule (mainly included 10 mg ginsenoside Rg3 in one capsule) was supplied from Yatai Pharmaceuticals Co., Ltd. (Jiutai, China). The chemical structures of the analytes are depicted in Figure 1. Methanol (MeOH, HPLC grade) and acetonitrile (ACN, HPLC grade) was obtained from Thermo Fisher Scientific (Fairlawn, NJ, USA). Ammonium acetate was obtained from Zhiyuan Chemical Co., Ltd. (Tianjin, China). Formic acid (HPLC grade) was provided from Aladdin Co., Ltd. (Shanghai). Ultrapure water was acquired from the Milli-Q water purification system (Millipore, Bedford, MA, USA).

### 2.2. Animals

Male Sprague-Dawley rats, weighing 200–220 g, were obtained from Beijing Vital River Laboratory Animal Technology (Beijing, China). The rats were housed in an environmentally controlled breeding room (12 h light/dark cycle) for three days before the experiment, and the standard laboratory food and water were available *ad libitum*. All experimental protocols were approved by the First Affiliated Hospital of Zhengzhou University Animal Ethics Committee. Animal facilities and welfares were performed strictly in accordance with the National Institutes of Health Guidelines regarding the principles of animal care.

### 2.3. Preparation of Stock Solution, Calibration Standards, and Quality Control Samples

Dissolving accurately weighed amounts of standard osimertinib and nilotinib in methanol to obtain the stock solutions in a final concentration of 2 mg/mL. To prepare the working solutions, stock solutions were diluted with ACN/water (1/1, V/V). The calibration standard samples were prepared by spiking the corresponding working solutions with blank rat plasma. Final calibration standard concentrations for osimertinib were 400, 300, 200, 100, 50, 20, 5, and 1 ng/mL, respectively. In a similar way, LLOQ and QC samples were prepared in diluting working solutions with rat plasma at four concentration levels: 350 ng/mL (high quality control (HQC)), 30 ng/mL (medium quality control (MQC)), 3 ng/mL (low quality control (LQC)), and 1 ng/mL (lower limit of quantification (LLOQ)). All stock and working solutions were stored at −80°C until further analysis.

### 2.4. Sample Preparation

Samples were extracted from rat plasma by protein precipitation. For this method, a total of 50 *μ*L of plasma sample (blank, calibration standards, LLOQ, and QCs) was mixed with 5 *μ*L of IS (600 ng/mL) solution. Then, 150 *μ*L ACN was added for protein precipitation. The tubes were closed and vortexed and mixed for 1 min. The samples were centrifuged at 14,000 rpm for 10 min at 4°C. Then, 100 *μ*L of supernatant was separated and transferred to fresh EP tubes and kept in the refrigerator prior to analysis. Finally, 5 *μ*L of the solution was injected into the UPLC-MS/MS system.

### 2.5. Chromatographic Separation and MS/MS Conditions

Osimertinib and IS were analyzed by an Exion LC Analytical System (AB Sciex, USA) coupled with a Qtrap 4500 mass spectrometer equipped with Turbo Ion Spray Interface (AB Sciex, USA). Separation of osimertinib and IS was achieved on a Phenomenex HPLC Kinetex C18 column (2.1 × 50 mm, 2.6 *μ*m) with a column heater kept at 40°C. Gradient elution was applied at a flow of 0.2 mL/min and performed by varying the proportion of solvent A (10 mM ammonium formate with 0.1% formic acid buffer) and solvent B (ACN) as follows: 0–0.8 min (5% B); 0.8–1 min (5–60% B); 1–5.5 min (60%B); 5.5–6 min (60–5%B), 6–8 min (5%B). The temperature of the autosampler was optimized at 4°C, and the injection volume was 5 *μ*L.

The Qtrap 4500 mass spectrometer equipped with Turbo Ion Spray interface operating in positive ESI mode (AB Sciex, USA) was selected for mass spectrometric detection. The multiple reaction monitoring (MRM) mode was acquired, and the MS spectrometry parameters were defined as follows: source temperature 500°C; ion spray voltage 4500 V; nebulizer gas (gas1) 50 psi; heater gas (gas2) 50 psi; curtain gas 40 psi; a low collision gas. The dwell time for osimertinib and nilotinib was 100 ms. The mass-dependent parameters are summarized in Table 1, and the data acquisition was processed with Analyst™ software (AB Sciex, version 1.6.3, USA).

### 2.6. Analytical Method Validation

According to the guidelines of the US Food and Drug Administration (FDA) [15], European Medicines Agency (EMA) [16], CFDA guidelines for the validation of bioanalytical methods [17], and Pharmacopoeia of the People's Republic of China [18], the present UPLC-MS/MS method was validated by the following parameters: selectivity, linearity and LLOQ, precision and accuracy, matrix effect and recovery, and stability.

#### 2.6.1. Selectivity and Specificity

To explore the selectivity and specificity of this method, six individual blank rat plasma samples, blank rat plasma samples spiked with osimertinib and IS at LLOQ level, and actual rat plasma samples after oral administration of osimertinib were analyzed.

#### 2.6.2. Linearity and LLOQ

The linearity, expressed by calibration curves with the correlation coefficient (*R*^2^) and weighed 1/*x*^2^ quadratic least-squares regression analysis, was evaluated by analyzing the standard rat plasma samples over a range of 1–400 ng/mL (at least eight concentration levels) in three separate days. We defined the LLOQ as the lowest validated concentration on the calibration curve with a signal-to-noise ratio (S/N) of more than 10. The back-calculated concentration on this calibration curve was less than 15% of the nominal concentration and less than 20% at the LLOQ level.

#### 2.6.3. Precision and Accuracy

Four different concentration levels of QC samples (1, 3, 30, and 350 ng/mL for LLOQ, LQC, MQC, and HQC, resp.) with six replicates for osimertinib were determined to evaluate the precision (intraday and interday) and accuracy of this method. The intraday experiment was assayed in one run within one day, and the interday experiment was assessed in three analytical runs on three separate days. The intraday and interday precision were assessed by calculating the relative standard deviation (RSD) and accuracies, which was required to be within ±15% of the nominal concentrations for LQC, MQC, and HQC samples and should be less than 20% RSD for LLOQ. Accuracy was calculated by analyzing the averaged measurements to normal values, which was expressed in relative error percentage: [(calculated concentration − true concentration/true concentration) ^*∗*^ 100].

#### 2.6.4. Matrix Effect and Recovery

The matrix effect of osimertinib was investigated by matrix factor, a peak area ratio of the analyte/IS with three QC concentrations in the presence of matrix ions (rat plasma) to those in the absence of matrix at equivalent concentrations. The RSD% of the matrix effect should less than 15%. The recovery was assessed by comparing the peak areas obtained from extracted QC samples with those in the mobile phase at the same concentrations and expressed as percentage. The matrix effect and recovery of IS were evaluated in the similar method at a concentration of 600 ng/mL in plasma.

#### 2.6.5. Stability

*(1) Short-Term Stability.* QC samples (low, medium, and high levels) were assessed by analyzing QC samples kept at room temperature for six hours.

*(2) Autosampler Stability.* QC samples (low, medium, and high levels) were evaluated after the processed QC samples were placed in an autosampler (4°C) for 24 hours.

*(3) Freeze-Thaw Stability.* QC samples (low, medium, and high levels) were investigated after storage at room temperature for 8 h, followed by three freeze-thaw cycles (thawing at room temperature during 2 h and freezing again at −80°C for at least 12 h).

*(4) Long-Term Stability.* QC samples (low, medium, and high levels) were determined by analyzing after storage at −80°C for 28 days.

### 2.7. Application to Pharmacokinetic Study in Rats

The rats were randomly divided into two groups (six rats for each group). Osimertinib was dissolved and suspended by 1% carboxymethylcellulose sodium solution. The Rg3 powder in Shenyi capsule was dissolved in saline. In the first period, ginsenoside Rg3 (5 mg/kg) was orally administered to the rats in group I for seven days, and the rats in the other group were orally treated with normal saline for seven days. In the second period, the rats were fasted overnight with free access to water before the experiment. After overnight fasting, ginsenoside Rg3 (5 mg/kg) plus osimertinib (10 mg/kg) was orally administered to the rats in group I, and the rats received oral ginsenoside Rg3 30 min prior to osimertinib at the eighth day. Rats in group II were only orally treated with osimertinib (10 mg/kg) at the eighth day. Blood samples (each 100 *μ*L) were collected via the oculi chorioideae vein at 0.5, 1, 2, 3, 4, 5, 6, 7, 8, 10, 12, 15, and 24 h after treatment in heparinized tubes. The blood samples were immediately centrifuged at 3000 rpm for 10 min. The plasma was separated from blood samples and transferred to clean tubes and immediately stored at −80°C until further analysis. The plasma concentrations of osimertinib were determined according to the aforementioned developed UPLC-MS/MS method.

### 2.8. Data Analysis

The pharmacokinetic parameters, including the maximal plasma concentration (*C*_max_), area under the plasma concentration-time curve (AUC), time for the maximal plasma concentration (*T*_max_), and mean residence time (MRT), were calculated through noncompartmental analysis by Phoenix WinNonlin (Pharsight Inc., USA, version 1.1) software.

All data were presented as the mean ± SD. Statistical differences analyses between the mean values were performed in SPSS software version 11.5 (SPSS, Chicago, IL, USA) and analyzed for significance by a nonpaired two-tailed Student's t-test. *p* values less than 0.05 were considered statistically significant.

## 3. Results and Discussion

### 3.1. Optimization of Mass Spectrometric Parameters

**Figure 2:**
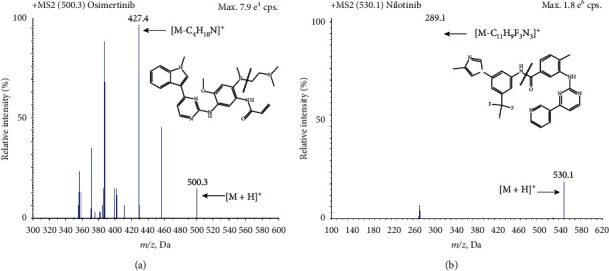
Product ion mass spectra of osimertinib (a) and nilotinib (b) in positive mode and their proposed fragmentation patterns.

In order to achieve high sensitivity and better response, the positive ionization and multiple reaction monitoring scan mode was applied and optimized by a systematic approach. The precursor and product ions were determined by directly injecting standard solution of osimertinib and IS to the mass spectrometer (Figure 2). It was observed that the high-intensity peaks of the analytes were conducted in these optimized mass spectrometric conditions. The main mass spectrometer parameters of osimertinib in our study were similar to previous studies [19, 20].

### 3.2. Optimization of Chromatographic Conditions

To optimize the appropriate chromatographic conditions for separation, commercially available columns and various mobile phases (various proportions and gradients) were evaluated for their chromatographic behavior and the ionization response.

The results indicated that Phenomenex HPLC Kinetex C18 column (2.1 × 50 mm, 2.6 *μ*m) achieved reasonable run time, symmetric peak shape, and good resolution. The different proportions of ACN were chosen on account of its low background noise, strong elution effect, and narrower peaks. Various proportions and gradients of water, 0.1% formic acid, and ACN and water, 1, 2, 5, and 10 ammonium formate, and ACN were tested during optimization. The results indicated that ammonium formate (10 mM) with 0.1% formic acid in gradient elution achieved desired separation, good peak symmetry, retention time, and appropriate MS responses. The retention time for osimertinib and nilotinib (IS) was 2.51 and 2.90 min, respectively, in a runtime of 8.00 min.

### 3.3. Sample Preparation

Various extraction was evaluated to achieve a maximum extraction efficiency of osimertinib and IS in rat plasma. The solid-phase extraction method was unnecessary for its complication. Poor recovery was observed in liquid-liquid extraction method with *n*-hexane and ethyl acetate as extraction solvent. As previously mentioned, the protein precipitation method was applied for its simple steps and sufficient recovery. The methanol and ACN were tested for protein precipitation solvents, and the results implied that cold ACN could achieve high protein precipitation efficiency and better mass spectrometric response. A sample: ACN ratio of 1 : 4 resulted in a promising recovery of osimertinib and IS. One hundred *μ*L of rat plasma was prepared for sample preparation as Dong described [14]. Many blood samples from each rat were collected for the pharmacokinetic study, which may influence the physiological and functional characteristics of rats. In addition, the supernatant was evaporated to dryness through nitrogen gas for sample preparation which was time-consuming and tedious [14]. Protein precipitation method was chosen in our experiment, and the method was simple and fast.

### 3.4. Analytical Method Validation

#### 3.4.1. Selectivity and Specificity

**Figure 3:**
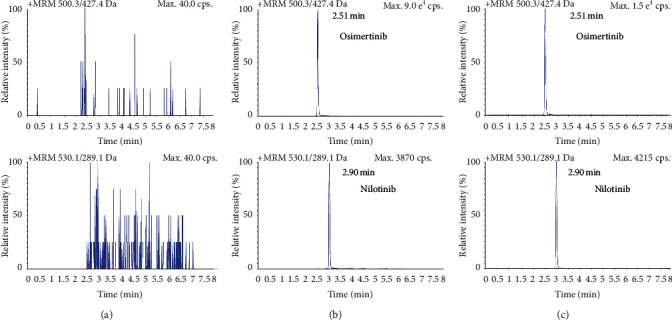
Typical MRM chromatograms of osimertinib and nilotinib (IS). (a) Blank plasma matrix sample; (b) blank plasma matrix sample spiked with the analytes (at medium concentrations); (c) real plasma sample obtained from rat after intravenous administration of osimertinib.

There was no significant chromatographic interference peaks from endogenous substances at the retention time with the osimertinib and IS in rat plasma. The responses of osimertinib in blank rat plasma samples were all <20% of the LLOQ response, and the blank IS responses were below 1% of the normal response. The typical chromatograms of blank rat plasma, blank plasma sample spiked with osimertinib and IS at LLOQ level, and rat plasma sample after administration were shown in Figure 3.

#### 3.4.2. Linearity and LLOQ

Good linearity of the calibration curve of osimertinib, generated by linear regression of peak area ratios against concentrations with a weighting factor of 1/*x*^2^ at eight concentrations over the range of 1 to 400 ng/mL in rat plasma, was observed in this method. The regression coefficients of all the calibration curves were greater than 0.99, and the typical equation was *y* = 0.04111*x*  + 0.02013 (*r*^2^ = 0.99862) (y represents the peak area ratios of osimertinib to the IS; *x* stands for the plasma concentrations of osimertinib). Back-calculated concentrations of calibration standards for osimertinib in rat plasma are listed in Table 2. LLOQ for determination of osimertinib in rat plasma was 1 ng/mL, and the ratio of signal-to-noise was much higher than five.

#### 3.4.3. Accuracy and Precision

The intra- and interday precision and accuracy for the determination of osimertinib at LLOQ and three QC levels in rat plasmas on three consecutive days are summarized in Table 3. Compared with nominal concentration, RSD of LLOQ and QC concentration were less than 15%, and the accuracy was within 85%–115%. The intra- and interday precision and accuracy assays only investigated LQC, MQC, and HQC concentrations as Dong described [14]. According to the Bioanalytical Method Validation Guidance of FDA, the precision and accuracy assays should be validated with four QC levels per run (LLOQ, LQC, MQC, and HQC) and should be ±20% of nominal concentration at LLOQ [15]. The four QC levels (LLOQ, LQC, MQC, and HQC) were validated for precision and accuracy assay in our experiment. Overall, the accuracy and precision values were within the validation criterion, and the method was highly reliable and reproducible for the determination of osimertinib in rat plasma.

#### 3.4.4. Matrix Effect and Extraction Recovery

The ratio of the peak area in the presence of matrix to the peak area in absence of matrix was determined as matrix factor. The IS-normalized osimertinib was calculated by dividing the osimertinib of the analyte by the osimertinib of the IS. As shown in Table 4, the RSD of the IS-normalized matrix factor of osimertinib and IS in rat plasma at three QC levels was in the range of 0.71% to 6.97%, which demonstrates that no endogenous substance could cause a significant effect on ionization. The overall mean extraction recovery of osimertinib in rat plasma was 99.72 ± 7.11% to 109.75 ± 8.38%, and the recovery of IS was 98.99 ± 7.90%, indicating no significant loss during the extraction process. As previously mentioned, the results indicated that high recovery and no significant matrix effect aided the successful validation of the method for osimertinib and IS in rat plasma.

#### 3.4.5. Stability

The stability of osimertinib at three QC concentrations under four different storage conditions is described in Table 5. According to the results, the measured concentrations, kept at room temperature for 6 h, placed in an autosampler for 24 h, freeze-thawed (−80°C) for three cycles, and stored at −80°C for 28 days, were all within ±15% of the norminal concentrations, which indicated that osimertinib was stable under the conditions investigated in this study.

#### 3.4.6. Effect of Rg3 on the PK Profiles of Osimertinib in Rats

**Figure 4:**
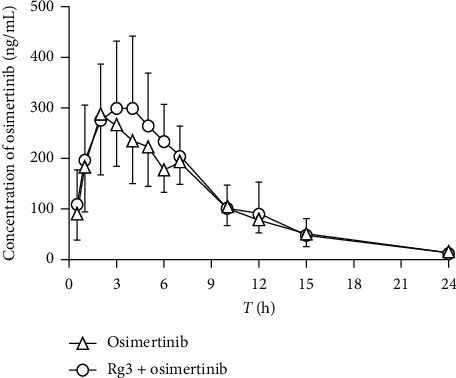
Mean plasma concentration–time profiles of osimertinib in male rats (each point represents mean ± SD). (A) Mean plasma concentration–time profiles of osimertinib after intravenous administration at a single dose of 10 mg/kg in male rats (*n* = 10); (B) mean plasma concentration–time profiles of osimertinib after intravenous administration in presence of Rg3 (5 mg/kg, 7 days) in male rats (*n* = 6).

The current fully validated UPLC-MS/MS method was successfully applied for determining the concentration of osimertinib in rat plasma after oral administration of osimertinib (10 mg/kg) to male rats in the presence or absence of Rg3 (5 mg/kg) for seven days. The mean plasma concentration–time curves of osimertinib in rats are shown in Figure 4, and the major pharmacokinetic parameters are summarized in Table 6.

The pharmacokinetic data showed that osimertinib was slowly absorbed at the highest plasma concentration in a long time (*T*_max_ was 3.33 ± 0.82 h) after oral administration (10 mg/kg) in rats. The peak plasma concentration (*C*_max_) was 0.317 ± 0.138 *μ*g/mL achieved with the area under the curve (AUC_0-*t*_) that was 2.823 ± 1.206 h·*μ*g/mL. The mean terminal half-life (*t*_1/2_) was 4.46 ± 0.94 h, and the mean residence time (MRT_0-*t*_) was estimated to be 6.88 ± 0.62 h. Additionally, the plasma clearance (CL/F) and distribution volume (V_z_/F) values were 19.821 ± 8.132 L/h/kg and 129.695 ± 58.112 L/kg, respectively. All the calculated pharmacokinetic parameters revealed that osimertinib gets absorbed slowly into blood and exerts a moderate elimination rate in rats. The obtained pharmacokinetic parameters were in agreement with the previous studies [13, 14].

Small-molecule tyrosine kinase inhibitors (TKIs) that target the EGFR are commonly used in combination with Chinese herbal medicines (e.g., ginseng, astragalus, and *Scutellaria barbata*) to enhance the efficacy and overcoming the drug resistance in NSCLC patients [21, 22]. Osimertinib was given frequently coadministered with many compound preparations including active ingredient Rg3 for exerting antihepatitis, anti-inflammatory, and antiviral effects. It is worth noting that the potential for clinical DDIs with these EGFR-TKIs could provide recommendations for managing, minimizing, or avoiding DDIs with the different agents [3, 23]. A clear and detailed understanding of their propensity for drug–drug interactions (DDIs) is required before their concomitant use in NSCLC patients. However, there was no report regarding the effect of Rg3 on the pharmacokinetics profile of osimertinib. As Rg3 and osimertinib were usually taken long term in clinic, we have investigated the effect of Rg3 on the pharmacokinetics profile of osimertinib for the first time.

On coadministering with Rg3 (5 mg/kg) for seven days, *C*_max_, AUC_0-*t*,_*t*_1/2_ values of osimertinib in rats were 0.297 ± 0.122 *μ*g/mL, 2.597 ± 0.839 h·*μ*g/mL, 5.08 ± 1.03 h, respectively. There were no significant changes observed in *C*_max_, AUC_0-t_, *t*_1/2_, *T*_max_, CL/F, and the other main pharmacokinetic parameters of osimertinib in coadministered and alone group. The results implied that there might have been no significant effect on the main pharmacokinetic parameters of osimertinib in rats when coadministered with Rg3 (5 mg/kg) for seven days. It is reasonable to hypothesize that Rg3 may have no potential effect on CYP3A in rats because of CYP3A mainly mediated the metabolism of osimertinib.

This validated UPLC-MS/MS method and pharmacokinetic study may prove to be of great significance for future investigations when conducting human clinical drug interaction trials on Rg3 and osimertinib.

## 4. Conclusions

In conclusion, a sensitive, rapid, and selective UPLC-MS/MS method was developed and validated for quantification of osimertinib in rat plasma, within a linear range of 1–400 ng/mL. This method was successfully applied to the pharmacokinetic study of osimertinib in rats across different administrations. This is the first study that discloses the effect of Rg3 on the pharmacokinetic profiles of osimertinib in rats at a clinical dosage level. Pharmacokinetic properties demonstrated that osimertinib exhibited a slow absorption and moderate-rate elimination in rats following oral administration of osimertinib (10 mg/kg). Coadministration with Rg3 (5 mg/kg) for seven days may have no obvious effects on the pharmacokinetics of osimertinib in rats. This study provides the theoretical foundation for the concomitant medications of Rg3 and osimertinib such that Rg3 could improve the life span, quality of life, and survival rate of NSCLC patients.

## Figures and Tables

**Table 1:** General mass spectrometric settings for the osimertinib and internal standards.

Analytes	*m*/*z* (Q1 ⟶ Q3)	ESI	RT (min)	DP (V)	EP (V)	CE (V)	CXP (V)
Osimertinib	*m*/*z* 500.3 ⟶ *m*/*z* 427.4	+	2.51	95	10	34	16
Nilotinib (IS)	*m*/*z* 530.1 ⟶ *m*/*z* 289.1	+	2.90	150	10	40	7

**Table 2:** Calibration standards for osimertinib in rat plasma (linear weighted 1/*x*^2^, *n* = 6).

Analytes	Item	Nominal standards concentrations (ng/mL)								QCs (ng/mL)			*R* ^2^
		S1 (1)	S2 (5)	S3 (20)	S4 (50)	S5 (100)	S6 (200)	S7 (300)	S8 (400)	L (3)	M (30)	H (350)	
Osimertinib	Mean	0.98	4.85	20.61	48.82	93.82	218.05	293.07	405.60	2.96	31.29	361.65	0.9972
	RSD (%)	5.61	6.06	9.68	3.39	5.95	6.84	0.79	3.65	8.48	1.71	4.75	0.12
	RE (%)	−2.31	−3.01	3.05	−2.36	−6.18	9.02	−2.31	1.27	−1.27	4.28	3.33	

**Table 3:** Intraday and interday precision and accuracy of osimertinib in rat plasma.

Analytes	Spiked concentration (ng/mL)	Intraday (*n* = 6)			Interday (*n* = 18)		
		Mean ± SD (ng/mL)	RSD (%)	Accuracy (%)	Mean ± SD (ng/mL)	RSD (%)	Accuracy (%)
Osimertinib	1 (LLOQ)	1.02 ± 0.11	10.4	102.5	0.99 ± 0.10	10.3	98.5
	3 (QC-L)	2.94 ± 0.20	6.9	98.1	2.91 ± 0.20	6.8	97.0
	30 (QC-M)	31.0 ± 0.5	1.7	103.2	30.3 ± 1.6	5.2	101.1
	350 (QC-H)	368 ± 11	3.1	105.1	365 ± 13	3.5	104.3

**Table 4:** Matrix effect and extraction recovery of osimertinib in rat plasma (*n* = 6).

Analytes	Spiked concentration (ng/mL)	Matrix effect		Extraction recovery	
		Mean ± SD (%)	RSD (%)	Mean ± SD (%)	RSD (%)
Osimertinib	3 (QC-L)	105 ± 7	7.0	108 ± 2	1.7
	30 (QC-M)	109 ± 6	5.7	100 ± 7	7.1
	350 (QC-H)	114 ± 1	0.7	110 ± 8	7.6
IS	600	—	—	99.0 ± 7.9	8.0

**Table 5:** Stability of osimertinib in rat plasma under various storage conditions (*n* = 6).

Spiked concentration (ng/mL)	Short-term (room temperature for 6 h)			Autosampler (4°C for 24 h)			Freeze-thawing (−80°C for 3 cycles)			Long-term (−80°C for 28 days)		
	Mean ± SD (ng/mL)	RSD (%)	RE (%)	Mean ± SD (ng/mL)	RSD (%)	RE (%)	Mean ± SD (ng/mL)	RSD (%)	RE (%)	Mean ± SD (ng/mL)	RSD (%)	RE (%)
3	3.20 ± 0.14	4.2	6.8	3.02 ± 0.01	0.4	0.6	3.03 ± 0.02	0.8	1.0	2.96 ± 0.24	8.2	−1.5
30	30.1 ± 1.0	3.1	0.4	28.7 ± 0.8	2.7	−4.2	30.1 ± 1.0	3.1	0.4	32.7 ± 0.8	2.3	9.1
350	341 ± 3	0.8	−2.7	359 ± 26	7.1	2.5	349 ± 6	1.6	−0.3	359 ± 7	2.0	2.4

**Table 6:** Pharmacokinetic parameters of osimertinib following oral administration (10 mg/kg) to male rats in the presence or absence of Rg3 (5 mg/kg, 7 days, *i.g.*, *n* = 6 for each).

PK parameter	Osimertinib alone	Rg3 + Osimertinib
*C*_max_ (*μ*g/mL)	0.317 ± 0.138	0.297 ± 0.122
*T*_max_ (h)	3.33 ± 0.82	2.60 ± 0.55
*t*_1/2_ (h)	4.46 ± 0.94	5.08 ± 1.03
AUC_0-*t*_ (h·*μ*g/mL)	2.82 ± 1.20	2.60 ± 0.84
AUC_0-∞_ (h·*μ*g/mL)	2.90 ± 1.21	2.72 ± 0.90
MRT_0-t_ (h)	6.88 ± 0.62	7.18 ± 0.75
CL/F (L/h/kg)	19.8 ± 8.1	20.0 ± 6.2
V_z_/F (L/kg)	130 ± 58	146 ± 54

Values are mean ± SD.

## Data Availability

The data used to support the findings of this study are included within the article.

## Abbreviations

NSCLC: Non-small cell lung cancer
EGFR-TKI: Epidermal growth factor receptor tyrosine kinase inhibitor
UPLC-MS/MS: Ultra-performance liquid chromatography-tandem mass spectrometry
CXP: Collision cell exit potential
DP: Declustering potential
EP: Entrance potential
CE: Collision energy
IS: Internal standard
MRM: Multiple reaction monitoring
ESI: Electrospray ionization
LLOQ: Lower limit of quantification
ME: Matrix effect
CMC-Na: Sodium carboxymethylcellulose
QC: Quality control
ULOQ: Upper limit of quantification
RSD: Relative standard deviation
RE: Relative error
ACN: Acetonitrile.

## References

[1] Jänne P. A., Yang J. C.-H., Kim D.-W. (2015). AZD9291 in EGFR inhibitor-resistant non-small-cell lung cancer. *New England Journal of Medicine*.

[2] Carlisle J. W., Ramalingam S. S. (2019). Role of osimertinib in the treatment of EGFR-mutation positive non-small-cell lung cancer. *Future Oncology*.

[3] Xu Z.-Y., Li J.-L. (2019). Comparative review of drug-drug interactions with epidermal growth factor receptor tyrosine kinase inhibitors for the treatment of non-small-cell lung cancer. *OncoTargets and Therapy*.

[4] Vishwanathan K., Dickinson P. A., So K. effect of itraconazole and rifampicin on the pharmacokinetics of osimertinib. *British Journal of Clinical Pharmacology*.

[5] Kim P., Su W., Miao Z.-h., Niu H.-r., Liu J., Hua Q.-l. (2008). Effect and mechanism of ginsenoside Rg3 on postoperative life span of patients with non-small cell lung cancer. *Chinese Journal of Integrative Medicine*.

[6] Guo X.-w., Hu N.-d., Sun G.-z., Li M., Zhang P.-t. (2018). Shenyi capsule plus chemotherapy versus chemotherapy for non-small cell lung cancer: a systematic review of overlapping meta-analyses. *Chinese Journal of Integrative Medicine*.

[7] Li N., Wang D., Ge G., Wang X., Liu Y., Yang L. (2014). Ginsenoside metabolites inhibit P-glycoprotein in vitro and in situ using three absorption models. *Planta Medica*.

[8] Liu Y., Zhang J.-W., Li W. (2006). Ginsenoside metabolites, rather than naturally occurring ginsenosides, lead to inhibition of human cytochrome P450 enzymes. *Toxicological Sciences*.

[9] Malati C. Y., Robertson S. M., Hunt J. D. (2012). Influence of Panax ginsengon cytochrome P450 (CYP)3A and P-glycoprotein (P-gp) activity in healthy participants. *The Journal of Clinical Pharmacology*.

[10] Zhang J., Zhou F., Wu X. (S)-ginsenoside Rh2 noncompetitively inhibits P-glycoprotein in vitro and in vivo: a case for herb-drug interactions. *Drug Metabolism and Disposition*.

[11] Hao L.-Q., Wang B., Gan H. Enhanced oral bioavailability and anti-tumour effect of paclitaxel by 20(s)-ginsenoside Rg3in vivo. *Biopharmaceutics & Drug Disposition*.

[12] Dou S. I., Cho M. K. (2013). The amelioration of N-acetyl-p-benzoquinone imine toxicity by ginsenoside Rg3: the role of Nrf2-mediated detoxification and Mrp1/Mrp3 transports. *Oxidative Medicine and Cellular Longevity*.

[13] Xiong S., Deng Z., Sun P., Mu Y., Xue M. (2017). Development and validation of a rapid and sensitive LC-MS/MS method for the pharmacokinetic study of osimertinib in rats. *Journal of AOAC International*.

[14] Dong S.-T., Li Y., Yang H.-T. accurate and effective method for measuring osimertinib by UPLC-TOF-MS and its pharmacokinetic study in rats. *Molecules*.

[15] Food and Drug Administration (FDA) (2013). *Draft Guidance for Industry in Bioanalytical Method Validation*.

[16] European Agency for the Evaluation of Medicinal Products (EMEA) (2011). *Guideline on Bioanalytical Method Validation*.

[17] China Food and Drug Administration, 2005, http://www.sda.gov.cn/gsz05106/08.pdf

[18] National Pharmacopoeia Committee (2015). *Pharmacopoeia of the People’s Republic of China*.

[19] Rood J. J. M., van Bussel M. T. J., Schellens J. H. M., Beijnen J. H., Sparidans R. W. (2016). Liquid chromatography-tandem mass spectrometric assay for the T790M mutant EGFR inhibitor osimertinib (AZD9291) in human plasma. *Journal of Chromatography B*.

[20] Zheng X., Wang W., Zhang Y. (2018). Development and validation of a UPLC-MS/MS method for quantification of osimertinib (AZD9291) and its metabolite AZ5104 in human plasma. *Biomedical Chromatography*.

[21] Zhang Z., Qi F., Cui Y. (2018). An update on Chinese herbal medicines as adjuvant treatment of anticancer therapeutics. *BioScience Trends*.

[22] Zhang X.-W., Liu W., Jiang H.-L., Mao B. (2018). Chinese herbal medicine for advanced non-small-cell lung cancer: a systematic review and meta-analysis. *The American Journal of Chinese Medicine*.

[23] Kucharczuk C. R., Ganetsky A., Vozniak J. M. (2018). Drug-drug interactions, safety, and pharmacokinetics of EGFR tyrosine kinase inhibitors for the treatment of non-small cell lung cancer. *Journal of the Advanced Practitioner in Oncology*.

